# Disease Progression of Idiopathic Cervical Dystonia in Spite of Improvement After Botulinum Toxin Therapy

**DOI:** 10.3389/fneur.2020.588395

**Published:** 2020-11-12

**Authors:** Harald Hefter, Isabelle Schomaecker, Max Schomaecker, Sara Samadzadeh

**Affiliations:** Department of Neurology, University Hospital of Düsseldorf, Düsseldorf, Germany

**Keywords:** cervical dystonia, natural history, progression of CD, botulinum toxin therapy, long-term outcome, secondary treatment failure, neutralizing antibodies (NAB)

## Abstract

**Aim of the Study:** To demonstrate general progression of symptoms in cervical dystonia (CD) on the one hand and improvement of some special symptoms on the other hand after botulinum toxin (BoNT) therapy.

**Methods:** 74 patients with idiopathic CD under continuous treatment in a BoNT outpatient department with at least three injections, completed a short questionnaire. They were asked whether pain, increased muscle tone and tension, reduced mobility of the head, abnormal head position, head tremor, or other symptoms had been present at the onset of BoNT-therapy and which symptoms were present at the time of recruitment. Patients had to rate actual severity of CD in percent of the severity of CD at the onset of BoNT-therapy. The TSUI score was determined by the treating physician. Blood samples were taken to analyze induction of neutralizing antibodies.

**Results:** Mean improvement of CD reported by the patients and scored by the physician was about 50%. The frequency of all symptoms increased with duration of therapy. The symptom most frequently improved was abnormal head position. The longer the time span between onset of symptoms and onset of BoNT-therapy was, the higher was the actual TSUI score and the lower the improvement reported. Twelve patients had positive antibody tests.

**Conclusions:** Patients experience a progression of CD, but recognize improvement of abnormal head position due to BoNT-therapy. The longer patients have been without BoNT- therapy, the poorer is the long-term outcome independent on duration of BoNT treatment. Therefore BoNT-therapy should be initiated as early as possible.

## Introduction

Idiopathic focal cervical dystonia (CD) is a chronic neurological disorder that usually becomes clinically manifest at age 40–60 years, and affects women 1.5–1.9 times more often than men (1–3). It's prevalence is 3 to 8/100,000 people in Europe or the US (4, 5). The broad spectrum of clinical symptoms has been characterized in detail and ranges from mild elevated muscle tone in circumscribed head or neck muscle groups—which can lead to abnormal head postures—to severe involuntary jerks of head and neck muscles, impaired control of head coordination and involvement of additional muscle groups of face, extremities and trunk (2, 3, 6). The impairment of head control interferes with daily activities and reduces patient's quality of life (7–10).

Intramuscular injections of botulinum toxin type A (BoNT/A) improve abnormal head position of CD in up to 70% of the patients (11, 12), and have become treatment of choice for CD (13). Nowadays, only a small percentage of <20% of mildly affected CD-patients stays without BoNT treatment over a longer period of time (14, 15). Interestingly, in spite of the large extent of improvement due to BoNT injections most CD-patients combine BoNT therapy with additional therapies ranging from standard physiotherapy to acupuncture, hypnosis, meditation, bio resonance therapy, to yoga, prayer, and other religious practices (15). The percentage of patients using more than 10 therapies in parallel did not differ between patients receiving BoNT injections (66.7%) and those who did not (64.4%) (15). Obviously, CD-patients are not completely satisfied with their BoNT therapy (14, 16) and discontinue BoNT therapy in a fairly large percentage ranging between 30% (17) up to 46% (18) [for details and an overview see (19)]. The reasons for discontinuation of BoNT therapy are only partly understood (19).

The development of a more complex pattern of CD during the course of BoNT treatment in at least 17% of the patients may be a possible reason for dissatisfaction and cessation of therapy. When 78 patients were split up into a “good” effect (*n* = 52) and an “unsatisfactory” effect (*n* = 26) subgroup the proportion of patients who changed to more complex patterns was significantly higher in the “unsatisfactory” compared to the “good” effect group (14).

Little is known about the natural history of CD without BoNT intervention. Most information comes from the pre-BoNT aera. Spontaneous remissions seem to be rare (3–10%) and usually occur during the first 3–5 years after first onset symptoms (20, 21). In the following years symptoms seem to fluctuate, three different phases of the spontaneous course of CD have been described by neurosurgeons (22). Deterioration is observed during the first 5 years, stable condition during the next 5 years and possible improvement thereafter (22). Furthermore, development from focal to multifocal or segmental dystonia in up to 30% of CD-patients has been reported (21, 23), but generalization is a red flag for symptomatic dystonia (24).

Since BoNT treatment is not a causal but a symptomatic therapy of CD, we were interested to document whether those symptoms, which had been present when BoNT therapy was initiated, persisted, or whether they improved or worsened or whether new symptoms developed during BoNT treatment, indicating that CD may progress in spite of some improvement. Furthermore, we were interested to see whether there was a negative influence on long-term outcome of BoNT therapy of time to therapy since onset of symptoms as observed for the long-term outcome of patients with generalized dystonia after deep brain stimulation (DBS) (25).

## Methods

This monocentric, cross-sectional, observational study was performed according to the declaration of Helsinki and the guidelines for good clinical practice (GCP). All patients gave written informed consent.

### Patients and Treatment-Related Data

All patients in the BoNT outpatient clinic of HH (at the University of Düsseldorf; Germany) were screened and informed on the purpose of the study and. Inclusion criteria were: (i) age > 18 years, (ii) patient not under care, (iii) diagnosis of focal CD, (iv) BoNT/A therapy was started in the BoNT/A outpatient clinic of HH, and (v) continuous BoNT/A treatment on a regular basis every 3–4 months at least 3 times. Exclusion criterium was segmental and/or symptomatic dystonia. Seventy-four CD-patients were included.

At the day of recruitment all patients underwent a careful clinical neurological investigation. Severity of CD was scored by means of the TSUI score (26) by the treating physician about 3 months after the last injection just before the next injection (ATSUI). To save time in daily practice, the TSUI-score, but not the TWSTRS rating scale (27) is routinely used in our BoNT clinic. Preparation of BoNT/A and dose per session (ADOSE) were documented. TSUI-score at the day of onset of BoNT/A therapy (ITSUI) and preparation of BoNT/A and dose per session at onset of BoNT/A therapy (IDOSE) were extracted from the charts of the patients. For sake of comparison doses were converted into unified dose units (uDU) by leaving onabotulinum toxin type A (onaBoNT/A) and incobotulinumtoxin type A (incoBoNT/A) doses unchanged and dividing abobotulinumtoxin type A (aboBoNT/A) doses by three following evidence-based data and a European consensus recommendation (28). Increase of dose during BoNT/A therapy (INDOSE) was calculated as ADOSE-IDOSE. Date of onset of BoNT/A therapy and duration of BoNT/A treatment (DURT) were also extracted from the charts. Improvement of CD assessed by the treating physician (IMPTSUI) was calculated as (ITSUI-ATSUI)/ITSUI.

Alternative therapies (acupuncture, physiotherapy, etc.) were not controlled in the present observational study.

### Symptom Questionnaire

While patients were waiting to be injected they had to complete a questionnaire asking the patients when they had noticed symptoms of CD for the first time (= age at onset of symptoms; AOS) and at which age they had been injected with BoNT/A the first time (= age at onset of therapy; AOT). The time span without BoNT therapy from onset of symptoms to onset of therapy (DURS) was calculated. Age at recruitment was documented. Patients were asked for the percentage of improvement or worsening in % of the severity of CD at onset of BoNT/A therapy (IMPQ). Severity of CD at day of recruitment (ASCD) had to be marked on a visual analog scale (0–100). The difference 100-ASCD was used as a second assessment parameter of the improvement of CD during BoNT/A therapy (IMPD).

The questionnaire contained a list of symptoms: pain in shoulder and neck muscles and tendons (PAIN), elevated muscle tone and muscle spasms (TONE), reduced mobility of head and shoulders (RMOB), abnormal head position (HPOS), and head tremor (HTRE). Patients had to remember and mark those symptoms which had been present when they had been treated the first time with BoNT. Patients had the opportunity to add further symptoms (OTHS) as muscle jerks, myoclonus, etc. to the list under the heading “other symptoms.”

Thereafter the questionnaire including the “other symptoms” was presented a second time to the patients and patients had to mark those symptoms which were present at the day of recruitment.

Finally, the questionnaire including the “other symptoms” was presented a third time to the patients and patients had to mark which symptoms had disappeared or had come up as a new symptom during BoNT/A therapy. This allowed a cross-check of patients' answers.

### Antibody Testing

Blood samples were taken, coded and sent to an independent blinded contractor (Toxogen®, Hannover, Germany). The mouse hemidiaphragma test (MHDA) was performed, the test result per coded sample was sent back months after the recruitment of the patients into the present study.

### Statistics

A non-parametric rank correlation analysis of the following 12 parameters was performed (AGE, AOS, DURS, DURT, IDOSE, ADOSE, INDOS, ITSUI, ATSUI, IMPQ, IMPD, IMPTSUI). Only a selection of relevant correlations is presented in the results. The non-parametric Friedman-test was used to compare the distribution of the frequency of symptoms (PAIN, TONE, RMOB, HPOS, HTRE, OTHS) at the onset of therapy and day of recruitment. Statistical analyses were performed using the SPSS statistics package (version 25; IBM, Armonk, USA).

## Results

### Long-Term Outcome of CD

Patients had a typical gender distribution (49 females, 25 males; relation: 2:1) and had a typical age of onset (mean AOS: 45.3 years; S.D.: 12.1; range: 14.7–73.4 years). Mean AOT was 51.2 years (S.D.: 11.8; range: 25.4–73.5 years). Mean DURT was 9.9 years (S.D.: 6.9; range: 0.6–26.7 years).

At the time of investigation 8/74 (=10.8%) of the patients were treated with onaBoNT/A, 30/74 (=40.5%) patients with aboBoNT/A, and 36/74 (=48.6%) with incoBoNT/A. During the course of treatment in 21/74 (=28.4%) patients had been switch from the complex protein containing BoNT/A preparations Botox® or Dysport® to the complex protein free BoNT/A preparation Xeomin® because of suspected secondary treatment failure (STF). Mean IDOSE was 169 uDU (S.D.: 82.1; range 31–500 uDU), mean ADOSE was 218 uDU (S.D.: 110; range: 19–500 uDU).

Mean ITSUI was 9.1 (S.D.: 3.1; range: 3–15), mean ATSUI was 4.3 (S.D.: 2.58; range: 0–10). This is an improvement of 52.7% in the mean. There was no correlation between ITSUI-score and IDOSE (uDU): *r* = 0.1425; n.s.). A highly significant positive correlation was present between ATSUI and ADOSE (*r* =0.4891; *p* < 0.001). But the increase of dose (INDOSE) per patient since onset of therapy tended to be negatively correlated with DURT (*r* = −0.1754; n.s.). The correlation between ITSUI and ATSUI was negative and highly significantly correlated (*r* = −0.709; *p* < 0.01), whereas IMPTSUI was positively and highly significantly correlated with ITSUI (*r* = 0.601; *p* < 0.01).

**Figure 1:**
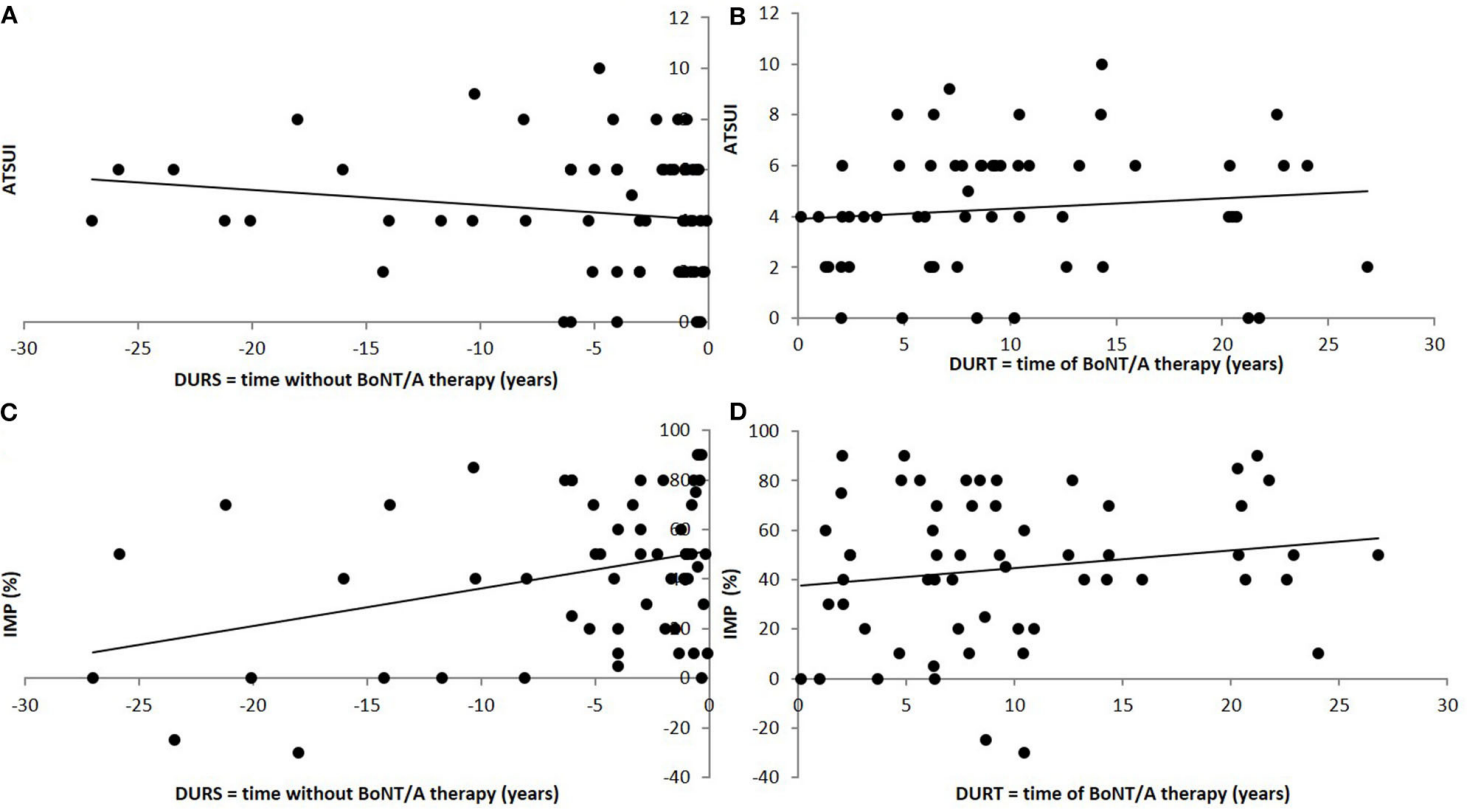
Correlation between long-term outcome and time to therapy **(A)** and duration of treatment **(B)**. **(A)** Significant (positive) correlation between the long-term outcome after BoNT/A therapy (ATSUI) and the time from onset of symptoms to onset of BoNT/A therapy (DURS). **(B)** No significant correlation between the long-term outcome after BoNT/A therapy (ATSUI) and the duration of BoNT/A therapy (DURT). There is a tendency to slightly higher values with duration of therapy. **(C)** Highly significant (negative) correlation between patient's estimation of improvement (IMP) and the time from onset of symptoms to onset of BoNT/A therapy (DURS). **(D)** Significant (positive) correlation between patient's estimation of improvement (IMP) and the duration of BoNT/A therapy (DURT).

Patients reported an improvement (IMPQ) of +45.9% in the mean (S.D.: 32.2; range: −30 to 100%; negative improvement indicates worsening). Mean actual severity of CD (ASCD) marked on a VAS (0–100%) was 53.9% (S.D.: 31.91; range: 2.2–130%) of the initial severity of CD at onset of therapy. IMPD was 46.1% and nearly identical to IMPQ. ASCD and ATSUI were significantly correlated (*r* = 0.324; *p* < 0.01), but not ASCD and ADOSE (*r* = 0.1516; n.s.). ATSUI did not significantly change with (DURT) (*r* = 0.1421; n.s.; Figure 1B), whereas IMPD significantly increased and ASCD significantly decreased with DURT (*r* = −0.4494; *p* < 0.001; Figure 1D). The correlation between IMPQ and IMPTSUI was one of the highest in the correlation matrix (*r* = 0.749; *p* < 0.0001).

### Long-Term Outcome in Dependance on preBoNT History

Mean DURS was 5.9 years (S.D.: 7.75; range: 0.1–36.6 years). No correlation between DURS and ITSUI was found (*r* = 0.15; n.s.), but ATSUI was positively and significantly correlated with DURS (*r* = 0.5090; *p* < 0.001; Figure 1A) indicating that a longer time to treatment was associated with a worse long-term outcome. This is consistent with a significant negative correlation between DURS and IMPQ (*r* = −0.300; *p* < 0.05; Figure 1C). IDOSE was not correlated with DURS (*r* = 0.0298; n.s.).

### Comparison of the Spectrum of Symptoms Before and After BoNT-Therapy

**Figure 2:**
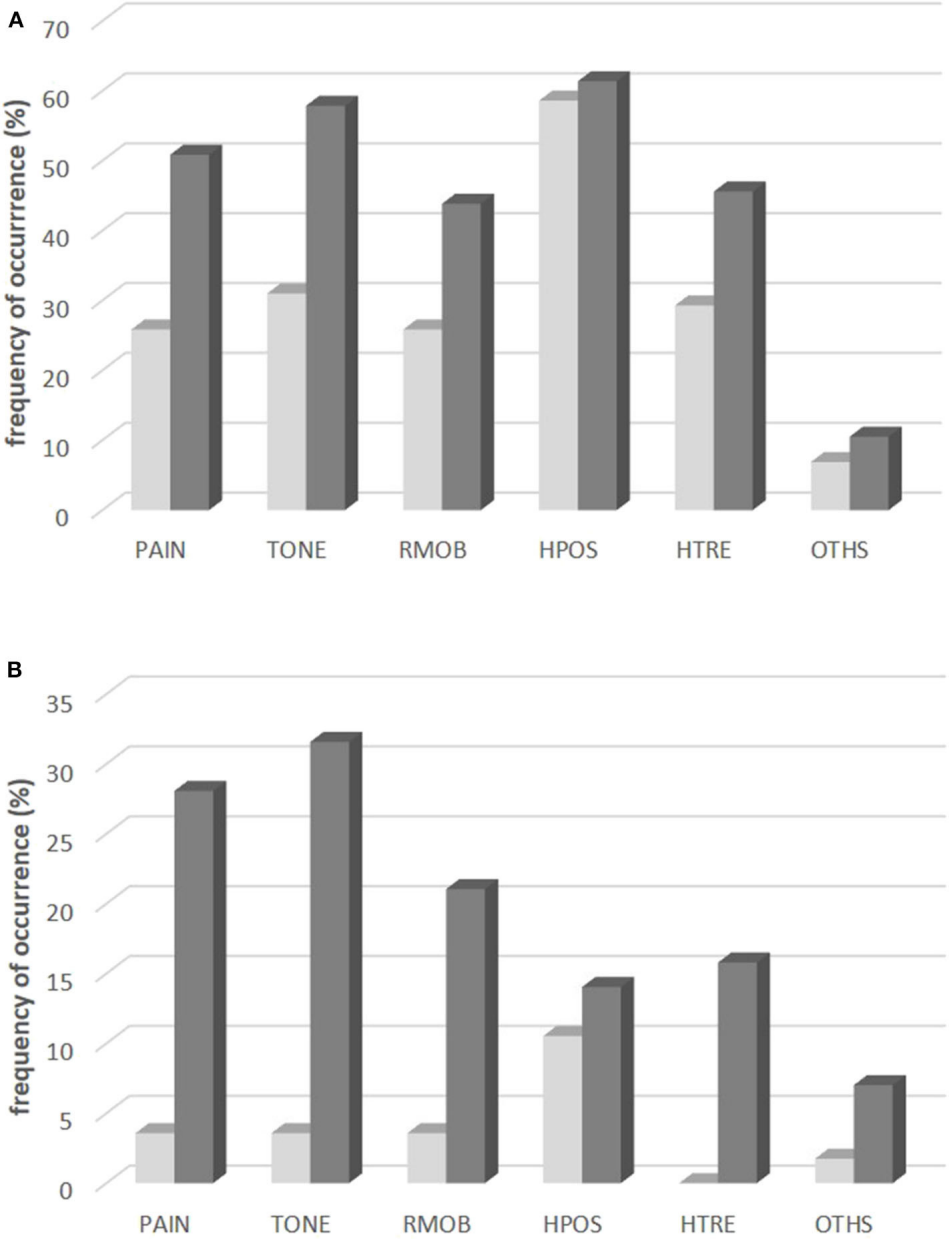
Frequency of occurrence **(A)** and changes **(B)** of six different symptoms of CD. **(A)** Frequency of occurrence of the symptoms PAIN, TONE, RMOB, HPOS, HTRE, OTHS in the entire population: at onset of BoNT/A therapy (light gray bars) and at the time of recruitment after BoNT/A therapy (dark gray bars). **(B)** Percentage of patients who reported disappearance of the symptoms PAIN, TONE, RMOB, HPOS, HTRE, OTHS during BoNT/A therapy (light gray bars) and percentage of patients who reported coming up of the symptoms PAIN, TONE, RMOB, HPOS, HTRE, OTHS during BoNT/A therapy (dark gray bars).

The by far most frequent symptom before BoNT therapy was abnormal head position (HPOS; 58%; see Figure 2A light gray bars). Elevated muscle tone (TONE; 31%) and head tremor (HTRE; 29%) were less frequently claimed. After BoNT/A therapy (see dark gray bars in Figure 2A) HPOS was still the most frequent symptom (61%), but elevated muscle tone (TONE; 57%), and pain (PAIN; 51%) had become manifest in much more patients. Thus the spectrum of symptoms had significantly (*p* < 0.001) changed during the treatment with BoNT/A.

Normalization of head position as a result of BoNT/A therapy was reported in 11% of the patients by the “golden responders.” Disappearance of PAIN, TONE, RMOB, and HTRE was reported by <5% of the patients (see light gray bars in Figure 2B). In about one third of the patients PAIN and elevated muscle tone (TONE) had developed as new symptoms during BoNT/A therapy (see dark gray bars in Figure 2B).

### Prevalence of MHDA-Positive Patients

Twelve out of 74 patients (=16.2%) were positive in the MHDA test. No difference of the spectrum of symptoms at recruitment and onset of BoNT/A therapy in the MHDA-positive and the MHDA-negative patients could be detected.

## Discussion

### Progression of Symptoms of CD During Long-Term BoNT/A Therapy

The present study demonstrates expansion of the spectrum of symptoms of CD during BoNT/A therapy. It has been reported previously that simple CD may progress to segmental dystonia in up to 30% of the CD-patients in the course of disease (23). Furthermore, an increase of the complexity of CD during BoNT treatment has been reported (14). When patients were split-up into an “unsatisfactory effect” and a “good effect” subgroup significantly more patients with a progressing CD were found in the “unsatisfactory effect” subgroup (14). Therefore, the expansion of the spectrum of symptoms in more than 30% of CD-patients reported in the present study nicely matches these previous reports. Additionally, it offers an explanation why some CD-patients may become unsatisfied with and may discontinue their BoNT/A treatment (19).

### Overestimation of the Treatment by the Treating Physician

This expansion of symptoms or progression of CD under continuous BoNT/A therapy does not imply that BoNT/A injections did not have any effect. Contrarily, patients recognized an improvement of CD of about 46% in the mean. Mainly head position was improved. About 10% of the patients were “golden responders” in whom head position had become normal (see Figure 2B, light gray bars). Treating physician's estimation of the treatment effect yielded a slightly better improvement of about 53% in the mean. This “overestimation” of the treatment effect in comparison to the patients has repeatedly been reported previously (10, 29) and reflects that the physician scores what he sees, but not what the patient feels and what he cannot rate as, e.g., the impact of CD on social life [for details see (10)]. Patients assessed the improvement of CD in general, the treating physician focused his scoring on special aspects of CD. Nevertheless, patient's and physician's rating of the treatment effect were highly significantly correlated.

### Efficacy of Long-Term BoNT Therapy

Intramuscular BoNT/A injections not only reduce muscle force and muscle tone, but also reduce the afferent feedback from the muscle spindles (30). Thereby intramuscular BoNT/A injections are a powerful tool to modulate the neck/shoulder muscle network and to reduce muscular hyperactivity. However, only part of the symptoms of CD is improved during BoNT/A therapy and the percentage of patients in whom symptoms disappear permanently is extremely low (Figure 2B). BoNT/A therapy is not a causal therapy for CD, BoNT/A injections do not cure the underlying disease process in the central nervous system.

Efficacy of BoNT therapy in the present study is comparable to other studies on long-term outcome. The higher the initial severity of CD the better the improvement and the lower the remaining severity of CD. The remaining severity of CD at the end of the last injection cycle [regardless whether assessed by the patients (ASCD) or scored by the treating physician by means of the TSUI-score (ATSUI)] was about 50% of the initial severity. This has been reported in previous studies from our center (11, 31) and from other centers (32, 33). In about 10% of the patients a complete normalization of head position was observed. But in an even larger percentage of patients abnormal head position developed as a new symptom (see Figure 2B). Most symptoms did not disappear during treatment, but in more than 1/3rd of the patients new symptoms showed up during BoNT/A therapy.

The majority of CD-patients is satisfied with their BoNT/A therapy. The adherence is high, at least in our center [for details see (34)], mean duration of therapy was 9.9 years. Injection techniques have been optimized, CD-patients were injected according the cap/col-concept since 2003 (35, 36). Nevertheless the spectrum of symptoms expanded, despite the well-documented significant improvement.

### Influence of the History of CD Prior to BoNT Therapy

It has been reported that CD usually progresses during the first 5 years after manifestation (22). Spontaneous remissions of CD seem to be rare (20, 21) but may occur during the first five years (22). During the course of CD degeneration of the cervical spine and herniations of discs can occur leading to radicular symptoms and compression of the cervical spine (25, 37). In the present study a significant negative correlation between time to therapy (DURS) and long-term outcome of BoNT/A therapy (ATSUI) was found: the longer the time to therapy the worse the long-term outcome. Obviously, the negative effect of the progression of CD prior to BoNT therapy cannot be compensated by BoNT injections later on completely. This negative influence of DURS could also be detected and was even more pronounced when patients' estimations of improvement (IMPQ) were correlated with DURS.

This matches the analysis of long-term outcome after DBS in patients with generalized dystonia. Among all factors influencing outcome of DBS of patients with primary generalized dystonia the duration of the disease prior to operation appears to be the only or most relevant factor (37, 38).

### Induction of NABs With Duration of BoNT Therapy

There was a mild, non-significant increase of the TSUI-score with duration of therapy (Figure 1B). In NAB-negative patients a mild continuous decrease of the TSUI-score has to be expected with duration of therapy (31) whereas in NAB-positive patients a significant increase of the TSUI-score with duration of therapy is found (31). In patients being treated for more than 10 years a rate of NAB-positive patients of at least 14% can be expected [for details see (31, 39)]. The percentage of 12 out of 74 MHDA-positive patients (=16.2%) in the present cohort, which was treated for 9.9 years in the mean, fits to this expectation. MHDA-positive patients have a higher severity of CD than MHDA-negative patients, are treated with higher doses and higher MHDA-titers are associated with more pain (40). In the present study the number of MHDA-positive patients was too small to detect differences in the distribution of symptoms between MHDA-positive and MHDA-negative patients.

## Conclusions

BoNT/A injection therapy effectively improves some symptoms of CD. Patients and treating physician score this treatment effect by about 50%. Nevertheless, BoNT/A is only a symptomatic therapy and cannot avoid the progression of CD and the appearance of new symptoms which had not been present at onset of BoNT/A therapy in more than 30% of the patients. This expansion of symptoms together with the negative impact of disease duration without BoNT/A therapy and the development of neutralizing antibodies probably contributes to patient's dissatisfaction and cessation of therapy. Because of a significant negative influence of the duration of the disease without therapy on long-term outcome BoNT/A therapy should be initiated as early as possible.

### Limitations of the Study

To some extent this study is based on remembered information provided by the patients on the presence of symptoms prior to BoNT therapy and development of symptoms during BoNT therapy. Therefore, a prospective study is recommended analyzing the symptoms of CD at onset of BoNT therapy and a couple of years later. But since it is unethical not to treat patients with BoNT therapy it will be difficult to find and to observe a sufficiently large sample of patients with CD over a sufficiently long period without BoNT therapy.

## Data Availability Statement

The raw data supporting the conclusions of this article will be made available by the authors, without undue reservation.

## Ethics Statement

The studies involving human participants were reviewed and approved by Heinrich-Heine University of Düsseldorf. The patients/participants provided their written informed consent to participate in this study.

## Author Contributions

IS and HH were involved in the design of the study, data collection, statistical analysis, preparation of the manuscript, and approval of the final draft. HH was treating physician for most of the patients and produced the first draft of the manuscript. MS and SS were involved in the statistical analysis. All authors have read and agreed on the content of the present version of the manuscript.

## Conflict of Interest

The authors declare that the research was conducted in the absence of any commercial or financial relationships that could be construed as a potential conflict of interest.

## Abbreviations

aboBoNT/A: abobotulinumtoxin type A (Dysport®)
ADOSE: total dose per session at day of recruitment
AGE: age at day of recruitment
AOS: age at onset of symptoms
AOT: age at onset of therapy
ASCD: patient's estimation of the severity of CD in percent of the severity at day of onset of BoNT/A therapy
ATSUI: TSUI-score at day of recruitment
BoNT: botulinum neurotoxin
BoNT/A: botulinum neurotoxin type A
CD: cervical dystonia
DURS: time span from onset of symptoms to BoNT/A therapy
DURT: duration of therapy
GCP: good clinical practice
HPOS: head position
HTRE: head tremor
IDOSE: first dose of BoNT/A
incoBoNT/A: incobotulinumtoxin type A (Xeomin®)
IMPD: patient's assessment of improvement by means of a visual analog scale
IMPQ: patient's assessment of improvement in percent of initial severity in the questionnaire
IMPTSUI: (ITSUI-ATSUI)/ITSUI^*^100
INDOSE: ADOSE-IDOSE
ITSUI: TSUI at onset of BoNT/A therapy
MHDA: mouse hemidiaphragma test
onaBoNT/A: onabotulinumtoxin type A (Botox®)
OTHS: other symptoms; to be added in the questionnaire
PAIN: pain
RMOB: reduced mobility of head; neck and shoulders
STF: secondary treatment failure
TONE: muscle tone in the neck muscles
TSUI: TSUI-score as introduced by the author TSUI
TWSTRS: Toronto spasmodic torticollis rating scale
uDU: unified dose units.
